# ASSESSING THE QUALITY OF CHATGPT RESPONSES TO DEMENTIA CAREGIVERS’ QUESTIONS POSTED ON SOCIAL MEDIA

**DOI:** 10.1093/geroni/igad104.2106

**Published:** 2023-12-21

**Authors:** Alyssa Aguirre, Robin Hilsabeck, Tawny Smith, Zhendong Wang, Ning Zou, Bo Xie, Daqing He

**Affiliations:** The University of Texas at Austin, Austin, Texas, United States; The University of Texas at Austin Dell Medical School, Austin, Texas, United States; The University of Texas at Austin, Austin, Texas, United States; University of Pittsburgh, Pittsburgh, Pennsylvania, United States; University of Pittsburgh, Pittsburgh, Pennsylvania, United States; The University of Texas at Austin, Austin, Texas, United States; University of Pittsburgh, Pittsburgh, Pennsylvania, United States

## Abstract

ChatGPT, an innovative, dialogue-based large-scale artificial intelligent model that responds to complex natural language inquiries, holds great promise to improve dementia caregivers’ quality of life by providing high-quality responses to meet their information needs. However, limited evidence exists on the quality of ChatGPT responses. This exploratory study aimed to address this gap. We first selected 25 social media posts, verified in our NIH-funded project (R56AG075770) as being representative of dementia caregivers’ needs for information about daily care for memory loss and confusion. We then collected ChatGPT responses in March 2023 and assessed their quality using a 4-item rating scale (1 point for each item; scoring range: 0-4; higher score indicates higher quality). This scale includes (1) Factual: Response contained no inaccurate or false information; (2) Interpretation: Response adequately interpreted the poster’s main need and correctly disregarded non-priority details; (3) Application: Response included both educational information and tangible actions; and (4) Synthesis: Response included follow-up actions (e.g., referrals). Three clinicians, each with 15+ years of experience with dementia caregivers, independently rated the responses. All raters agreed on the ratings for 22 (88%) responses initially; disagreements centered on responses’ comprehensiveness and specificity. After reconciliation, all raters agreed on the ratings: 3 (12%) responses received 3 points and 22 (88%) received 4 points, suggesting high quality. Study limitations included small sample, posts representing only one type of information wanted by caregivers, and potential ceiling effect of the rating scale. In our larger project, we will address these limitations and systematically assess ChatGPT responses.

